# Systemic administration of orexin A ameliorates established experimental autoimmune encephalomyelitis by diminishing neuroinflammation

**DOI:** 10.1186/s12974-019-1447-y

**Published:** 2019-03-20

**Authors:** Laurine Becquet, Catalina Abad, Mathilde Leclercq, Camille Miel, Laetitia Jean, Gaëtan Riou, Alain Couvineau, Olivier Boyer, Yossan-Var Tan

**Affiliations:** 1 University of Rouen Normandy, INSERM U1234 PANTHER, Institute for Research and Innovation in Biomedicine (IRIB), Faculté de Médecine et Pharmacie, 22 Boulevard Gambetta, 76183 Rouen, France; 2 Paris-Diderot University, INSERM U1149, Inflammation Research Center (CRI), DHU UNITY, Faculté de Médecine Site Bichat, 16 rue H. Huchard, 75018 Paris, France; 3 Department of Immunology and Biotherapy, University of Rouen Normandy, INSERM U1234 PANTHER, IRIB, Rouen University Hospital, 22 Boulevard Gambetta, 76183 Rouen, France

**Keywords:** Orexin A, Neuropeptide, Neuro-immunomodulation, Multiple sclerosis, EAE, Neuroinflammation

## Abstract

**Background:**

Orexins (hypocretins, *Hcrt*) A and B are GPCR-binding hypothalamic neuropeptides known to regulate sleep/wake states and feeding behavior. A few studies have shown that orexin A exhibits anti-inflammatory and neuroprotective properties, suggesting that it might provide therapeutic effects in inflammatory and neurodegenerative diseases like multiple sclerosis (MS). In MS, encephalitogenic Th1 and Th17 cells trigger an inflammatory response in the CNS destroying the myelin sheath. Here, we investigated the effects of peripheral orexin A administration to mice undergoing experimental autoimmune encephalomyelitis (EAE), a widely used model of MS.

**Methods:**

Mice were subcutaneously immunized with myelin oligodendrocyte glycoprotein peptide (MOG)_35–55_ in CFA. Mice were treated intraperitoneally for five consecutive days with either PBS or 300 μg of orexin A starting at a moderate EAE score. Molecular, cellular, and histological analysis were performed by real-time PCR, ELISA, flow cytometry, and immunofluorescence.

**Results:**

Orexin A strongly ameliorated ongoing EAE, limiting the infiltration of pathogenic CD4^+^ T lymphocytes, and diminishing chemokine (MCP-1/CCL2 and IP-10/CXCL10) and cytokine (IFN-γ (Th1), IL-17 (Th17), TNF-α, IL-10, and TGF-β) expressions in the CNS. Moreover, orexin A treatment was neuroprotective, decreasing demyelination, astrogliosis, and microglial activation. Despite its strong local therapeutic effects, orexin A did not impair peripheral draining lymph node cell proliferation and Th1/Th17 cytokine production in response to MOG_35–55_ in vitro.

**Conclusions:**

Peripherally-administered orexin A ameliorated EAE by reducing CNS neuroinflammation. These results suggest that orexins may represent new therapeutic candidates that should be further investigated for MS treatment.

**Electronic supplementary material:**

The online version of this article (10.1186/s12974-019-1447-y) contains supplementary material, which is available to authorized users.

## Introduction

Multiple sclerosis (MS) is a central nervous system (CNS) disease characterized by inflammation, demyelinating processes, and neurodegeneration. Evidence supports a Th1/Th17 autoimmune component driving chronic inflammatory processes in the spinal cord and brain. Thus, most MS therapies are immunomodulatory. However, they are only partially effective at early phases of the pathology with important side effects. In addition to inflammation, axonal and neuronal pathologies are major players during MS. Therefore, there is an urgent need to identify new therapeutic targets, capable not only of blocking inflammation but also of promoting axon integrity/regeneration, neuron survival, and remyelination.

Orexins A and B (hypocretins 1 and 2) are two neuropeptides of 33 and 28 aminoacids, respectively, which derive from a common precursor (prepro-orexin) [1]. They were initially identified by reverse pharmacology as the endogenous ligands for the orphan GPCRs, OX1R, and OX2R [2]. Whereas orexin A binds to both receptors with high affinity, orexin B only binds to OX2R with a similar high affinity [3]. Signaling pathways associated to orexin receptors are phospholipase A2, C and D, diacylglycerol lipase, Ca^2+^, and adenylyl cyclase cascades. As hypothalamic neuropeptides, orexins are known to regulate sleep and arousal states, appetite, feeding, and energy homeostasis [2]. Multiple studies have highlighted the therapeutic potential of the orexin system mainly in narcolepsy and other sleep disorders [4].

A few studies have suggested that orexin A exhibits anti-inflammatory properties. It was found that the endogenous orexin system attenuated brain damage in a murine focal cerebral ischemia model, an effect associated with reduced IL-6 and TNF-α [5]. In addition, peripheral administration of orexin A by a subcutaneous osmotic pump improved the survival of mice to lipopolysaccharide (LPS)-induced endotoxin shock, decreasing the levels of multiple proinflammatory cytokines and chemokines [6]. Recently, it has been shown that orexin A exhibited anti-inflammatory actions in a murine model of experimental colitis by inhibiting the NF-kB pathway [7]. In vitro studies using the microglial cell line BV2 suggest that orexin A may directly inhibit innate immune responses. In this sense, orexin A pre-treatment of BV2 cells decreased the production of proinflammatory IL-6, TNF-α, and iNOS upon toll-like receptor (TLR)-4 stimulation, while inducing an anti-inflammatory (M2-like) phenotype characterized by increased arginase-1 expression [8, 9].

Several reports demonstrated that orexin A also exerts neuroprotection, reinforcing its therapeutic potential in neurodegenerative diseases with an inflammatory component such as MS. Indeed, orexin A reduced brain damage in models of rodent cerebral ischemia [8, 10, 11]. In vitro, orexins A and B were protective against cobalt-induced oxidative stress in primary rat neuronal cultures [12]. Other investigators demonstrated protective effects of orexins on the SH-SY5Y human neuroblastoma cell line, an in vitro model of dopaminergic neurons in Parkinson’s disease, through anti-apoptotic and antioxidant effects [13, 14].

Using chronic MOG_35–55_-induced experimental autoimmune encephalomyelitis (EAE), a widely used model of MS, we investigated here the curative potential of peripheral orexin A administration in the clinical development of ongoing disease. Moreover, we studied the impact of this treatment on inflammatory and neurodegeneration processes that underlie the pathogenesis of EAE.

## Methods

### Mice

Animal experimentation guidelines dictated by the ethical committee for animal experimentation, CENOMEXA N°54 and by the French ministry (*Ministère de l*’*Education Nationale*, *de l*’*Enseignement Supérieur et de la Recherche*) were followed. All animals were 9–12-week-old female C57BL/6Rj mice (Janvier Laboratories, France) housed in an SPF-free vivarium with food and water ad libitum. Sets of *n* = 10 mice per group were used for clinical assessment until day 30 post-immunization. Separate sets of mice were EAE-induced for early (day 15, *n* = 6 mice/group) and late (day 21, *n* = 7 mice/group) sacrifices with the purpose of cellular and molecular analysis.

### EAE induction and orexin A treatment

EAE was induced by subcutaneous immunization in the flanks with 100 μg of MOG_35–55_ (GL Biochem, China) emulsified with complete Freund’s adjuvant (CFA) supplemented with 5 mg/mL of *Mycobacterium tuberculosis* H37Ra (Difco, USA). In addition, mice received intraperitoneally (IP) 300 ng of pertussis toxin (List Laboratories, USA) on days 0 and 2 post-immunization. Mice were scored daily from 0 to 5 by two researchers blinded to the treatments as described [15]: 0—no symptoms; 1—loss of tail tonicity; 2—wobbly gait; 3—partial paralysis of the two hind legs or complete paralysis of one hind leg; 4—complete paralysis of the two hind legs; 5—moribund state or death. Weight was assessed every other day during the course of the disease. All appropriate efforts were made to minimize animal suffering or discomfort, and to use the minimal number of animals necessary to produce reliable results. Mice were treated for five consecutive days with either PBS (control group), 100 μg or 300 μg of orexin A (GL Biochem) in 200 μl volume by IP or retro-orbital (RO) administrations. Treatment was initiated either in preventive (i.e., before disease onset = day 3 post-immunization) or curative (at a moderate EAE score = 1.5) settings. Mice were sacrificed by isoflurane overdose at the peak and chronic phases of the disease (days 15 and 21 post-immunization, respectively).

### Histopathology and immunofluorescence

Spinal cords were collected 21 days after immunization and fixed in 4% paraformaldehyde overnight. Seven-micrometer sections from paraffin-embedded tissues were prepared for hematoxylin-eosin and luxol fast blue staining. Sections were photographed at × 10 (Zeiss Axio Vert microscope) magnification, and image acquisition was performed with Zen 2 lite software. Histopathology was scored from 0 to 4 as follows: 0—no infiltration and no demyelination; 1—sparse immune cells in the meninges area; 2—few localized immune cell infiltrating areas reaching the parenchyma with mild demyelination; 3—multiple infiltration areas with moderate demyelination; 4—severe immune cell infiltration with severe demyelination. The percentage of demyelination was calculated on pictures containing the whole spinal cord section for each mouse. The percentage of demyelination was calculated with ImageJ (NIH) software. Colors were split in three channels and blue stained area measured and divided by the total spinal cord area.

For immunofluorescence studies, spinal cords were cryoprotected in 20% sucrose and embedded in OCT. Cryostat 7-μm sections were incubated with primary anti-CD4 (1:500, BD Biosciences, San Jose, USA), anti-MBP (1:1000, Millipore, Burlington, USA), anti-GFAP (1:1000, Thermofisher scientific, USA), anti-Iba1 (1:1000, Wako, Japan), or anti-Arg1 (1:200, Abcam, UK) antibodies overnight at 4 °C. Then, sections were incubated with Alexa Fluor 488 or Cy.3-conjugated secondary antibodies (1:400, Jackson ImmunoResearch Antibodies, West Grove, USA) for 45 min at room temperature. Slides were mounted using Fluoromount-G with DAPI (Southern Biotech, Birmingham, USA). Spinal cord sections were photographed at × 20 magnification on a Zeiss epifluorescence microscope (AxioImager J1) equipped with an apotome and Zen pro2012 software (two or three images per animal per antibody). A tiles tool was used to create images of full sections of each spinal cord, and measurements were performed on full spinal cord sections. Images were analyzed by an operator blinded to the treatment groups using ImageJ. Percentage of immunoreactive area or cell numbers per mm^2^ was quantified.

### Flow cytometry

Fifteen or 21 days post-immunization, lymph nodes were harvested and cell suspensions were obtained by shredding the organs through a 40-μm mesh. For myeloid cell analysis, cells were incubated with APC-Cy7-anti-CD3, APC-anti-CD11c, PerCP-Cy5.5-anti-Ly6C, BV605-anti-Ly6G, BV421-anti-PD-L1, FITC-anti-MHCII, and PE-Cy7-anti-CD86 from Sony (San Jose, USA) and Alexa700-anti-CD11b from eBioscience (San Diego, USA) for 30 min at 4 °C. For T cell proliferation, activation, and regulatory T cell (Treg) analysis, cells were incubated with APC-Cy7-anti-CD4, BV605-anti-CD8, PercP-Cy5.5-anti-CD62L, BV421-anti-CD69 (Sony), PE-anti-CD25 (BD Biosciences), and PE-Cy7-anti-CD44 (eBioscience) for 20 min at 4 °C. Then, cells were treated with fixation/permeabilization buffer (eBioscience) overnight, and incubated with APC-anti-Foxp3 (eBioscience) and FITC-anti-Ki67 (Sony) during 1 h. Samples were analyzed with a LSRFortessa cytometer (BD Biosciences) and FlowJo software.

### RNA extraction and real-time RT-PCR

RNA was extracted from spinal cords or axillary lymph nodes at day 15 and 21 post-immunization with Trizol (Sigma, Saint Louis, USA) and RNA retrotranscribed with iScript Reverse transcription supermix (Bio-rad, Hercules, USA). Real-time quantitative PCRs were performed using SYBR Green I Master (Roche) in combination with the specific primers (Additional file 1: Table S1). Amplification was performed with LightCycler 480 System SW 1.5.1 (Roche) as follows: initial denaturation during 10 min at 95 °C, 40 cycles of 96 °C for 20 s followed by 64 °C for 10 s and 72 °C for 15 s for IFN-γ, IL-17, and FoxP3; 96 °C for 20 s, 60 °C for 20 s, and 75 °C for 15 s for IL-4; 96 °C for 20 s, 60 °C for 30 s, and 72 °C for 20 s for IL-10; 96 °C for 30 s, 62 °C for 30 s, and 72 °C for 30 s for IFN-γ-inducible protein 10 (IP-10); 96 °C for 25 s, 60 °C for 35 s, and 72 °C for 35 s for monocyte chemoattractant protein 1 (MCP-1); 96 °C for 20 s followed by 60 °C for 10 s and 72 °C for 15 s for TGF-β and 96 °C for 20 s, 62 °C for 30 s, and 72 °C for 20 s for TNF-α. Melting curve analysis confirmed primer specificity. The calculation was normalized to the housekeeping gene HPRT according to the formula (*E*_target_)ΔC*t*_target_/(*E*_normalizer_)ΔCt_normalizer_ (Real-time PCR handbook from Life Technologies).

### Orexin receptor mRNA expression

For orexin receptor expression, spinal cord, brain, cervical and axillary lymph nodes, thymus, and spleen from naïve mice were collected. In addition, CD4^+^, CD8^+^, and CD11b^+^ cells were isolated from the spleen of naïve mice by using a FACSAria-IIIu cell sorter (BD Bioscience). Purity of isolated populations was above 95% (not shown). RNA was extracted with Trizol (Sigma), treated with DNase I (Invitrogen, Carlsbad, CA), and retrotranscribed by iScript reagent (Bio-Rad). PCRs were performed with Thermoprime Taq DNA polymerase (Thermofisher scientific) in combination with specific primers (Additional file 1: Table S1) in a thermocycler (Eppendorf, Hambourg, Germany).

### Antigen recall assays

On day 15 after immunization, cell suspensions from axillary lymph nodes were prepared as above. Cells were cultured at 1 × 10^6^ cells/mL in complete medium (RPMI 1640 containing 25 mM HEPES, 2 mM l-glutamine, 1% penicillin/streptomycin, and 10% FBS, Thermofisher scientific) with ovalbumin (Ova, Sigma) or MOG_35–55_ (20 μg/mL, GL Biochem) for 48 h. For some experiments, cells from PBS-treated mice were stimulated with Ova or MOG_35–55_ (20 μg/mL) in the presence or absence of orexin A (1 × 10^−6^ M). IFN-γ and IL-17 levels were measured by ELISA kits (Affymetrix, Santa Clara, USA) following the manufacturer’s protocol. For proliferation assay, after 48 h of culture, 1 μCi/well of [^3^H]-thymidine was added for 18 h. Incorporated radioactivity was measured on a Wallac 1450 Trilux β-scintillation counter.

### Statistical analyses

Statistical analysis was performed using Graphpad Prism 6.05 for Windows (La Jolla, USA). For statistical test of EAE score curves, two-way ANOVA was used. For the rest of analysis, Kruskal-Wallis (when multiple groups are compared) and Mann-Whitney (for comparison between two groups, i.e., EAE orexin-treated or non-treated) tests were used to assess significance. Significance threshold was determined at *P* < 0.05. Results were represented with the mean ± SEM.

## Results

### Systemic orexin A administration strikingly ameliorates the clinical features of ongoing chronic EAE

**Fig. 1:**
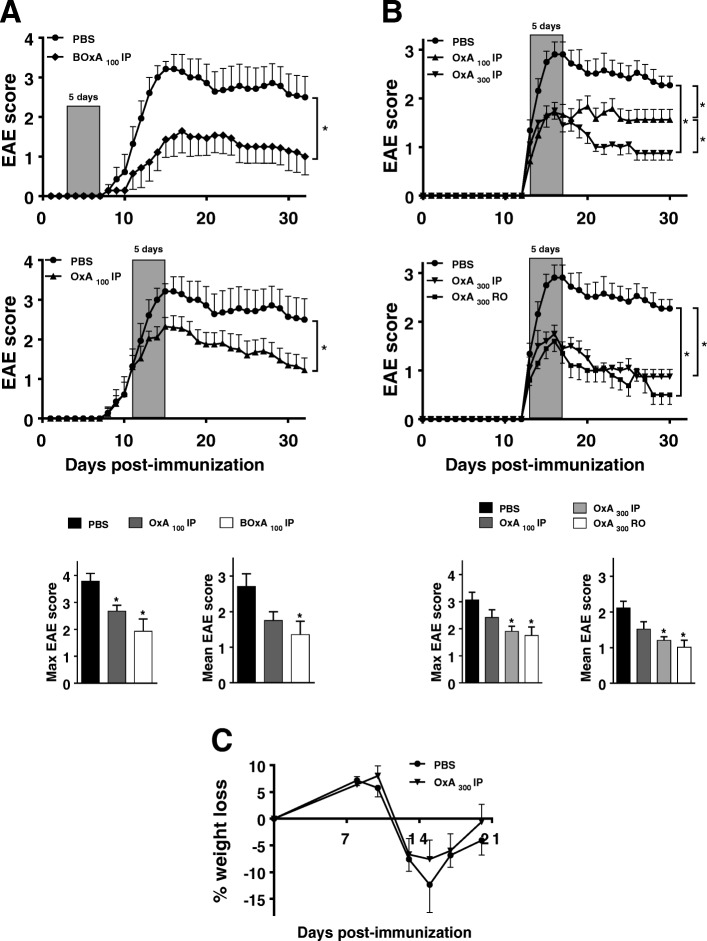
Preventive and curative orexin A treatments dramatically alleviate EAE symptoms. Chronic EAE was induced with 100 μg of MOG_35–55_ to 9-week-old female C57BL/6 mice (*n* = 10/group). Panels show clinical signs of EAE scored daily for over 30 days on a scale of 0–5 as described in the “Methods” section. **a** Shows intraperitoneally (IP) administered PBS (PBS group), 100 μg/mouse of orexin A for 5 days starting on day 3 (= before the onset, BOxA_100_ IP group) or starting when mice exhibit a moderate EAE score (= 1.5 for OxA_100_ IP group). **b** Shows IP given PBS (PBS group), 100 μg/mouse of orexin A (OxA_100_ IP group) or 300 μg/mouse of orexin A (OxA_300_ IP group), as well as retro-orbital (RO) injection with 300 μg/mouse of orexin A (OxA_300_ RO group) for a 5 day administration starting at moderate EAE (= 1.5). Clinical curves represent mean clinical scores ± SEM. *P < 0.05 (Two-way ANOVA compared to PBS group). For all clinical studies, maximal EAE score and mean EAE score were assessed. *P < 0.05 (Kruskal-Wallis test with Dunn’s post-hoc test for multiple comparisons with PBS group). **c** Shows the percentage of weight loss (in comparison with the initial weight before EAE induction) during the disease development. Shown results are representative of three independent experiments with *n* = 10 animals/group each

We investigated the therapeutic potential of systemic administration (i.e., IP or RO) of orexin A during EAE. First, we evaluated the effects of five IP injections of 100 μg/mouse/day of orexin A vs. PBS (control) on consecutive days in either preventive (starting on day 3 post-EAE induction, BOxA_100_ IP) or curative (start at EAE score of 1.5, OxA_100_ IP) settings (Fig. 1a). Both treatments efficiently ameliorated clinical EAE, as reflected by the reduction of the average maximal and mean scores (Fig. 1a). Because of the higher therapeutic interest of a curative vs. a preventive treatment from a clinical standpoint, the former was selected for further studies. The action of orexin A was dose-dependent, with an optimal effect observed with 300 μg/mouse/day (OxA_300_ IP) (Fig. 1b). A similar efficacy when orexin A was administered through retro-orbital route was found (Fig. 1b). Orexins regulate food intake and energy balance, however, body weight was not different between orexin A- vs. PBS-treated EAE mice along the study (Fig. 1c).

These results provide the proof-of-concept that orexin A exhibits potent therapeutic actions during EAE when given peripherally. Based on these data, we determined the optimal conditions (i.e., OxA_300_ IP), as well as two time points of sacrifice (i.e., day 15 as the peak and day 21 as the established phase of the disease) for further analyses.

### Orexin A treatment reduces CNS inflammation in EAE

**Fig. 2:**
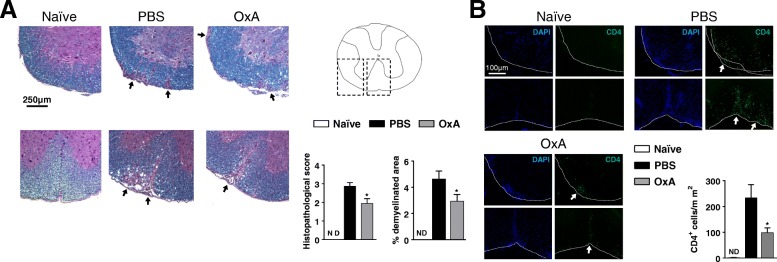
Orexin A significantly decreases the histopathological score of EAE mice and CD4^+^ T cell recruitment in the CNS. On day 21 post-immunization, spinal cord sections from naïve, PBS, and OxA_300_ IP mice were prepared. **a**:Spinal cord tissues were stained with hematoxylin/eosin (to distinguish cell infiltration) and luxol fast blue (to detect myelin). **b** Spinal cord sections were labeled with DAPI and Alexa 488 anti-CD4. All images were taken at × 20 magnification. Two or three representative areas of spinal cord are shown per experimental group. Arrows show inflammatory and demyelinating areas. Graphs show the mean histological scores and the quantification of infiltrated CD4^+^ cells per mm^2^ of each group (*n* = 7/group). *P < 0.05 (Mann-Whitney test, compared to PBS group)

**Fig. 3:**
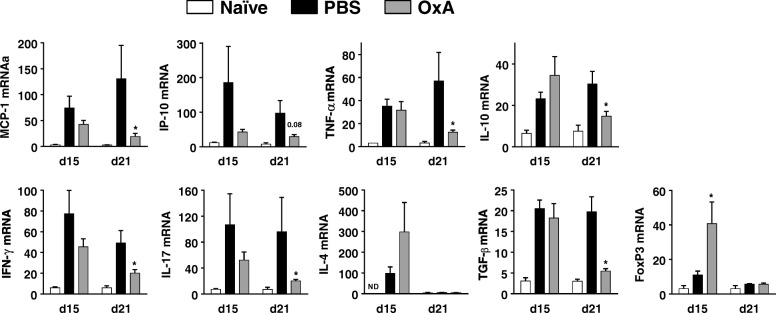
Orexin A significantly reduces inflammatory responses in the CNS. The panel shows the mRNA expression of chemokines (i.e., MCP-1 or CCL2 and IP-10 or CXCL10), cytokines (i.e., TNF-α, IL-10, IFN-γ (Th1), IL-17 (Th17), IL-4 (Th2), and TGF-β) and the transcription factor, Foxp3 (Treg-specific) determined by real time RT-PCR in naïve animals and EAE mice (i.e., 15 and 21 days after EAE induction in PBS- and OxA-treated mice, *n* = 6/group and n = 7/group respectively). *P < 0.05 (Mann-Whitney test, compared to PBS group)

The clinical beneficial effect of orexin A was associated to a significant reduction of the histopathological score on day 21 post-immunization (Fig. 2). Indeed, spinal cord sections from orexin A (vs. PBS) treated mice exhibited significantly reduced immune cell infiltration and myelin loss as determined by hematoxylin/eosin and luxol fast blue stainings, respectively (Fig. 2a). Indeed, we found by immunofluorescence staining that the number of infiltrating CD4^+^ cells infiltrating the meninges and CNS parenchyma was strikingly reduced in orexin A- mice vs. PBS-treated controls (Fig. 2b). These results suggest that orexin A impairs the recruitment of T cells to the CNS, critical for the development of neuroinflammation during EAE. Corroborating the lower degree of inflammation in the CNS of orexin A-treated mice, there was a significant generalized reduction of the expression of chemokines (MCP-1/CCL2, IP-10/CXCL10) and both proinflammatory (TNF-α, IFN-γ, IL-17) and anti-inflammatory cytokines (IL-10 and TGF-β), in the spinal cords of orexin A- compared to PBS-treated mice on day 21 post-EAE (Fig. 3). The expression of immune mediators was not different at the peak of EAE (day 15), except for Foxp3, a transcription factor that characterizes regulatory T cells (Tregs), which was significantly increased in orexin A-treated mice.

### Orexin A treatment decreases demyelination and astrocyte and microglial activation in the CNS during EAE

**Fig. 4:**
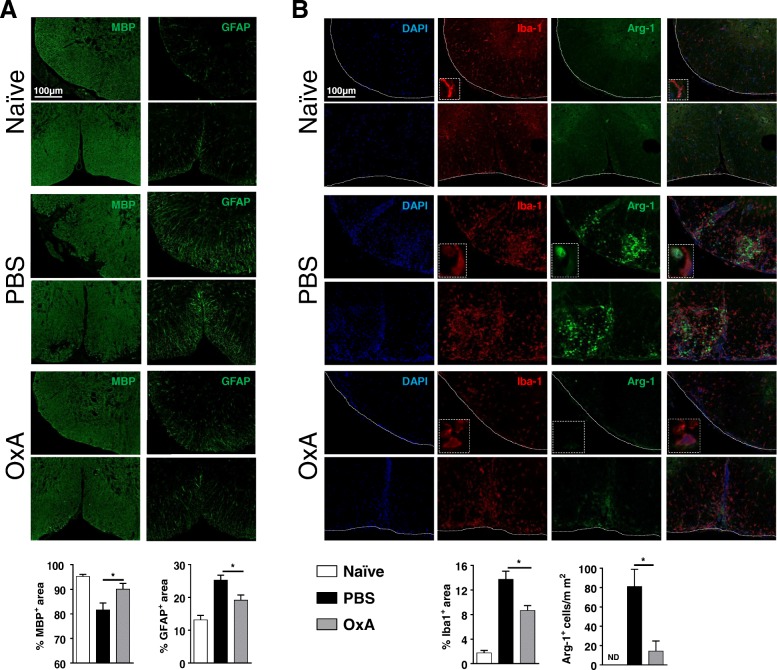
Orexin A strikingly diminishes demyelinating processes, astrogliosis, and microglial/macrophage responses in the CNS. On day 21 post-immunization, spinal cord sections from naïve, PBS, and OxA groups were prepared and labeled with **a** Alexa 488 anti-MBP or Alexa 488 anti-GFAP, and **b** DAPI, Cy3 anti-Iba-1, and Alexa 488 anti-Arg-1 stainings. All images were taken at × 20 magnification. Two representative areas of spinal cord are shown per experimental group. Graphs show the percentage of MBP, GFAP, and Iba1 immunoreactive areas as well as the Arg-1-positive cell number per mm^2^ of each group (*n* = 7/group). *P < 0.05 (Mann-Whitney test, compared to PBS group). *ND* not detectable

Demyelination is a hallmark of MS and EAE, and is strongly linked to the symptoms of the disease. By immunofluorescence staining of myelin basic protein (MBP) (day 21 post-EAE induction), we found that the percentage of MBP stained area in the white matter was significantly diminished in EAE vs. naïve mice, and that treatment of mice undergoing EAE with orexin A significantly reduced myelin loss (Fig. 4a).

In addition to the immune component of EAE pathogenesis, activated glial cells contribute to neurodegenerative processes. Astrocyte activation in the spinal cord occurred in response to EAE as reflected by the increase of percentage of stained GFAP^+^ area (Fig. 4a). A significant reduction in the percentage of GFAP^+^ area was found in orexin A- compared to PBS-treated mice, suggesting diminished astrogliosis.

Previous studies have shown that orexin A modulates microglial functionality in vitro. We used the marker Iba1 in order to study myeloid responses in EAE. In naïve mice, Iba1^+^ cells were broadly and evenly distributed in the spinal cord parenchyma (Fig. 4b). These cells were small and ramified, a morphology typical of resting microglia. In the spinal cords of EAE mice, there was a robust increase in the area of Iba1^+^ staining close to immune cell infiltration and demyelination sites (Fig. 4b). Furthermore, suggesting an activated phenotype, Iba1^+^ cells exhibited a round morphology with shorter processes than resting microglia in naïve animals (see inserts). Orexin A diminished the area of Iba1^+^ staining and thus reduced microglial activation and/or monocyte/macrophage infiltration (Fig. 4b). Because orexin A was found to increase in cultured microglia the expression of arginase-1 (Arg-1), an enzyme that has been ascribed to the M2 anti-inflammatory phenotype, we performed Iba1 and Arg-1 double staining. All Arg-1^+^ cells were Iba1^+^ and were abundant in EAE mice. However, their numbers were strongly reduced in orexin A-treated EAE mice (Fig. 4b). This further proves that orexin A blocks microglia activation and macrophage infiltration in EAE mice.

These results show that orexin A treatment protects from demyelination and may provide neuroprotection by reducing astrocyte and microglial cell activation, two events that are key in neurodegenerative processes.

### Orexin a receptors are expressed in the immune system

**Fig. 5:**
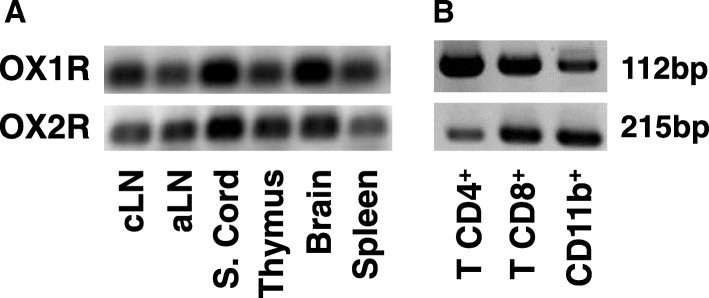
Orexin A receptors are widely expressed in the immune and nervous systems. The expression profile of the receptors OX1R and OX2R was determined in naïve animals by PCR. RNA was prepared and reversed transcribed from **a** immune (cervical/axillary lymph nodes (cLN and aLN, respectively), thymus, spleen) and CNS tissues (brain and spinal cord) and **b** different immune cell populations such as T CD4^+^, T CD8^+^, and CD11b^+^ splenocytes that were sorted by flow cytometry using the FACS Aria-IIIu (BD Bioscience). The results were assayed by electrophoresis

Our results suggest that orexin A has immunoregulatory properties, but the expression of orexin receptors in the immune system has not been described to date. We determined the mRNA expression of OX1R and OX2R in primary (thymus) and secondary (cervical, axillary lymph nodes, and spleen) immune organs of naïve mice by PCR. We found that both receptors are expressed in all immune tissues investigated (Fig. 5a). In addition, as previously described, orexin receptors exhibited a strong expression in the normal spinal cord and brain (Fig. 5a). We also studied the expression of OX1R and OX2R in FACS-sorted T lymphocytes (CD4^+^ and CD8^+^) and myeloid cells (CD11b^+^) from naïve animals (Fig. 5b). This revealed that all these immune cell types express both receptors, suggesting that they may directly respond to orexins.

### Orexin A treatment leads to an increase in myeloid and T cell numbers in the draining lymph nodes during EAE pathogenesis

The discovery of orexin receptors in immune cells in the periphery led us to study the impact of orexin A treatment in myeloid and T cell populations in the draining lymph nodes (DLNs) of EAE mice on day 21 post-immunization by flow cytometry. No major differences were found in myeloid cell proportions except for an increase of Ly6G^+^Ly6C^low^ cells and a decrease of Ly6G^−^Ly6C^high^ cells (Additional file 2: Figure S1). These phenotypes have been associated in some contexts with immunosuppression, receiving the names of granulocytic and monocytic myeloid derived suppressor cells (G-MDSCs and M-MDSCs), respectively [16]. In fact, we found lower levels of MHC class II and CD86, but higher PD-L1 in these cells than in CD11b^+^Ly6G^−^Ly6C^−^ cells (Additional file 2: Figure S1). This may suggest that these cells have either poor activating capacities or increased suppressive actions. Nevertheless, whether or not this result is associated to the beneficial effects of orexin A remains to be elucidated.

No significant differences were found regarding the proportions of T CD4^+^ and CD8^+^ cells (Additional file 3: Figure S2). We also investigated whether or not the orexin A treatment modulated the T cell proliferative/activation status. We found no differences in the proportions of proliferating (Ki67^+^), early activated (CD69^+^), naïve (Tn, CD44^−^CD62L^+^), effector memory (Tem, CD44^+^CD62L^−^), or central memory (Tcm, CD44^+^CD62L^+^) CD4^+^ and CD8^+^ T cells (Additional file 4: Figure S3). It has been shown that certain GPCR-binding neuropeptides such as vasoactive intestinal peptide (VIP), adrenomedullin and cortistatin, reduce inflammation in EAE by increasing the numbers of Tregs [17–19]. Tregs, identified as CD4^+^CD25^+^Foxp3^+^ cells, have suppressive activity on lymphocytes, and are critical for EAE recovery [20]. Orexin A treatment did not significantly alter the percentages of Tregs or their proliferative status (Additional file 3: Figure S2).

Although the results above show that the proportions of most myeloid and lymphoid cell subsets in the DLNs did not change upon orexin A treatment, we found that their total numbers were strikingly higher than in PBS-treated mice (Table 1). The accumulation of immune cells in the lymph nodes of orexin A-treated EAE mice might be linked to a diminished trafficking and infiltration to the CNS, in agreement with our histology findings.

**Table 1:** Orexin A treatment increases the total numbers of immune cells in draining lymph nodes during EAE

	Total cell numbers			
		Naïve	PBS	OxA
	DLN	4.86 ± 0.7 × 10^6^	9.46 ± 2.4 × 10^6^	26.97 ± 3.9 × 10^6^ *
Myeloid cells	CD11b^+^	1.96 ± 0.3 × 10^4^	19.44 ± 3.7 × 10^4^	57.37 ± 11.5 × 10^4^*
	CD11c^+^	3.96 ± 0.7 × 10^4^	7.13 ± 2.1 × 10^4^	32.94 ± 6.0 × 10^4^*
	CD11b^+^CD11c^+^	1.97 ± 0.3 × 10^4^	7.38 ± 2.1 × 10^4^	27.09 ± 4.5 × 10^4^*
	Ly6G^+^Ly6C^low^	3.92 ± 1.1 × 10^4^	5.24 ± 0.9 × 10^4^	22.65 ± 5.1 × 10^4^*
	Ly6G^−^Ly6C^high^	0.31 ± 0.08 × 10^4^	9.59 ± 1.9 × 10^4^	22.61 ± 4.6 × 10^4^*
CD4^+^ cells	CD4^+^	1.63 ± 0.2 × 10^6^	2.15 ± 0.4 × 10^6^	5.51 ± 0.8 × 10^6^ *
	Ki67^+^	8.92 ± 1.3 × 10^4^	33.41 ± 8.8 × 10^4^	95.39 ± 14.6 × 10^4^ *
	CD69^+^	8.93 ± 1.2 × 10^4^	4.26 ± 1.2 × 10^4^	9.61 ± 4.1 × 10^4^
	CD44^+^CD62L^+^ (Tcm)	11.47 ± 1.7 × 10^4^	23.97 ± 5.8 × 10^4^	57.31 ± 8.4 × 10^4^ *
	CD44^+^CD62L^−^ (Tem)	9.83 ± 1.0 × 10^4^	149.7 ± 28.1 × 10^4^	394.7 ± 64.9 × 10^4^ *
	CD44^−^CD62L^+^ (Tn)	137.0 ± 20.0 × 10^4^	34.21 ± 8.5 × 10^4^	80.32 ± 12.84 × 10^4^ *
	Tregs	16.10 ± 1.9 × 10^4^	28.88 ± 6.7 × 10^4^	72.92 ± 10.8 × 10^4^ *
	Ki67^+^Tregs	8.20 ± 1.0 × 10^4^	10.73 ± 2.7 × 10^4^	26.48 ± 4.1 × 10^4^*
CD8^+^ cells	CD8^+^	1.36 ± 0.2 × 10^6^	1.87 ± 0.4 × 10^6^	4.28 ± 0.7 × 10^6^ *
	Ki67^+^	61.54 ± 9.0 × 10^4^	16.59 ± 4.2 × 10^4^	45.13 ± 6.5 × 10^4^ *
	CD69^+^	6.16 ± 0.8 × 10^4^	4.23 ± 1.3 × 10^4^	8.34 ± 3.3 × 10^4^
	CD44^+^CD62L^+^ (Tcm)	20.69 ± 2.5 × 10^4^	34.0 ± 10.5 × 10^4^	70.94 ± 14.7 × 10^4^
	CD44^+^CD62L^−^ (Tem)	1.16 ± 0.1 × 10^4^	144.2 ± 26.7 × 10^4^	333.5 ± 53.8 × 10^4^ *
	CD44^−^CD62L^+^ (Tn)	112.8 ± 16.8 × 10^4^	3.43 ± 0.7 × 10^4^	9.24 ± 1.4 × 10^4^*

On day 21 after MOG-immunization, draining lymph node cells from naive, PBS, and OxA groups were analyzed by flow cytometry (*n* = 7/group). Table shows the absolute numbers of each subpopulation (i.e. myeloid cells, CD4^+^, and CD8^+^ cells). *P < 0.05 (Mann-Whitney test, compared to PBS group)

### Cell proliferation and Th1/Th17 responses upon antigenic challenge in vitro are not altered in EAE mice treated with orexin A

**Fig. 6:**
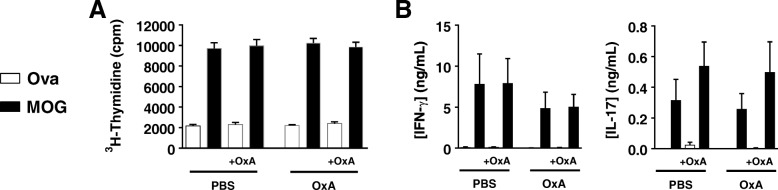
Orexin A does not interfere MOG-induced proliferation and cytokine release in lymph node cultures from PBS- and OxA-treated EAE mice. At the peak of the disease, on day 15 post-MOG immunization, draining lymph node cells from PBS- and OxA-treated mice were stimulated ex vivo with 20 μg/mL of MOG_35–55_ peptide or ovalbumin (Ova) in the presence or absence of OxA (1 × 10^−6^ M). **a** Shows cell proliferation that was measured by [^3^H]-thymidine incorporation. **b** Shows proinflammatory cytokine (i.e., IFN-γ and IL-17) secretion into the culture supernatant measured by ELISA. In both groups, all parameters measured were. No statistical differences were found between PBS and Orexin A treatments (Mann-Whitney test)

Priming of T cells against MOG_35–55_ in the DLNs is critical for EAE development. Whereas the data above show that orexin A treatment did not alter the proportions of different T cell populations, it does not reveal whether or not it modulates their responsiveness to MOG_35–55_. To address this question, we performed antigen-recall studies with DLN cells collected at the peak of the disease, and stimulated with MOG_35–55_ or the irrelevant antigen ovalbumin in vitro. Cells from both PBS- and orexin A-treated mice exhibited robust and equivalent proliferation and cytokine IFN-γ (Th1) and IL-17 (Th17) productions in response to MOG_35–55_ (Fig. 6). Moreover, these responses were not altered when orexin A was added to the cultures from PBS-treated mice. Confirming the lack of orexin effect on peripheral Th responses, the mRNA levels of IFN-γ, IL-17, and IL-4 (Th2), as well as the chemokine receptors CCR6 and CXCR3 were not different in non-stimulated DLNs cells isolated from PBS or orexin A treated mice (Additional file 5: Figure S4). These results suggest that the therapeutic actions of orexin A do not rely on an impairment of peripheral encephalitogenic Th responses.

## Discussion

This is the first demonstration that a systemic delivery of orexin A ameliorates EAE in preventive and curative settings. Interestingly, orexin A had a long-term beneficial effect in clinical disease (i.e., maintained until day 30), despite being administered for only 5 days, reinforcing its interest from a therapeutic standpoint. The amelioration of EAE clinical scores by orexin A was associated to a significant blockade of neuroinflammatory processes in the spinal cord.

Systemically delivered orexin A effects may modulate local (CNS) and/or peripheral immune responses. We found orexin receptor expression in both CNS and immune tissues of naïve mice. A previous report described an upregulation of hypothalamic orexin receptor mRNA expression upon EAE induction in the CNS [21]. The same team reported that a long term (day 2 to day 21 post-immunization) local CNS delivery of orexin A by intracerebroventricular (ICV) administration attenuated the disease [22]. ICV delivery of drugs to human patients is often unfeasible, diminishing its interest from a therapeutical standpoint. Whereas IP-delivered orexin A might easily reach immune organs, a direct action at the level of the CNS would require that this neuropeptide crosses the blood–brain barrier (BBB). In this sense, one study demonstrated that intravenously delivered orexin A but not orexin B was capable of crossing the BBB from the blood by simple diffusion [23]. Another study however claimed that orexin A cannot cross an intact BBB [24]. Whereas these studies were performed in basal conditions, in the context of inflammation which affects the permeability of this barrier, peripherally administered orexin A was capable of crossing the BBB and reach the CNS upon systemic LPS administration [6]. Therefore, in the EAE model of CNS inflammation where the BBB is disrupted [25] (as it is the case in MS patients [26]), both actions at CNS and peripheral levels might be, in principle, possible.

In EAE, encephalitogenic T cells against a myelin antigen are generated in the DLNs close to the immunization site, acquiring proinflammatory Th1 and Th17 profiles [27]. Upon their migration to the CNS, they are reactivated and trigger a local and deleterious inflammatory response, characterized by the recruitment of immune cells, the production of proinflammatory cytokines and chemokines, the activation of resident glial cells, the loss of the myelin sheath, and ultimately, axonal damage and neurodegeneration [28]. We found that orexin A strongly reduced the numbers of CD4^+^ cells infiltrating the spinal cord at the chronic phase of the disease, and that total numbers of T cells were increased in the lymph nodes of these mice. The peripheral accumulation of these cells might be a consequence of the lack of immune cell trafficking to the CNS rather than increased proliferation. The latter was not altered by orexin treatment as assessed by flow cytometry analysis of Ki67 staining and ex vivo antigen-recall assays. In correlation with the decrease in T cell infiltration, orexin A treatment led to a reduction of spinal cord chemokine expression (i.e., MCP-1 and IP-10), as well as a global reduction in proinflammatory cytokine mRNA levels. Fatemi et al. (2016a) reported that orexin A delivered ICV increased CNS TGF-β expression levels. This cytokine has been associated to anti-inflammatory responses in certain contexts. In our study, TGF-β levels were decreased as for the rest of cytokines analyzed, in agreement with a global inhibition of the immune response in the CNS. This discrepancy may be explained by the different administration methods (i.e., local vs. systemic) and the duration of the treatment (i.e., 20 vs. five days), that may lead to different mechanisms of action.

In addition to downregulating inflammation, orexin A diminished neurodegenerative processes including demyelination and astrocyte and microglial activation. Multiple studies have shown a central pathological role for astrocytes in neuroinflammation and particularly in EAE. Astrocytes have been recently shown to be activated in response to Th1 and Th17 cytokines [29]. Astrocyte activation by Th1 cells seems to be critical for EAE development, since silencing IFN-γ binding/signaling in these cells was shown to ameliorate EAE by downregulating the inflammatory cascade [30]. Interestingly, astrocytes are a major source of two IFN-γ-inducible chemokines, IP-10 and MCP-1, which promote lymphocyte and monocyte recruitment, respectively [31, 32]. Selective genetic invalidation of IP-10 or MCP-1 in astrocytes led to reduced EAE supporting their relevance for the amplification of the inflammatory response in the CNS [31, 32]. In agreement with the decrease of glial fibrillary acidic protein (GFAP) staining found upon orexin A treatment, the reduction in the expressions of IP-10 and MCP-1 suggests a decrease in astrocyte activation which may contribute to the amelioration of EAE.

Along with astrocytes, myeloid cells in the CNS such as resident microglial cells and infiltrating macrophages play an important role in the development of the disease. As a source of proinflammatory cytokines and chemokines, they can further amplify the recruitment of immune cells. However, it is well-known that these myeloid cells can also produce anti-inflammatory factors which contribute to the resolution of such immune response. Pro- and anti-inflammatory myeloid phenotypes are often referred to as M1 and M2 and can be induced in vitro upon exposure to specific cytokines [33]. Initial work demonstrated that orexin A induced in vitro microglial M2 polarization with increased arginase 1 [9]. In agreement with other studies, we found increased Iba1-positive cells expressing Arg-1 in EAE mice [34, 35]. Nevertheless, orexin A strongly reduced the presence of these cells. A recent study using LysM-EGFP reporter mice has reported that arginase 1 is only expressed by infiltrating myeloid cells in EAE [36]. Thus, the decrease in Arg-1-positive cells in our study may correlate with the reduced immune cell infiltration in orexin A-treated animals.

Our analysis of Th profiles in ex vivo experiments suggest that orexin A does not impair the generation of encephalitogenic Th cells in the periphery, but rather disrupt their recruitment to the CNS or their engagement of local proinflammatory responses in the CNS. In addition to the reduction in Th1 and Th17 cytokines, we found an increase of the mRNA expression of Foxp3, a transcription marker typically expressed by Tregs was found in the CNS only on day 15. Tregs are capable of suppressing T effector functions, and their expansion has been associated to the recovery from EAE [37]. Therefore, this finding could be associated to orexin beneficial actions in EAE.

Our study demonstrates a beneficial action of exogenously administered orexin A in the model of EAE. Whether or not the endogenous source of orexin is protective from developing MS remains to be elucidated. Some studies found reduced levels of orexin in the cerebrospinal fluid of MS patients [38–40]. Sleep disorders are common in MS patients, and a connection between narcolepsy and MS has been proposed. In fact, multiple lines of evidence have suggested that MS and narcolepsy/cataplexy may share common genetic traits [41]. A reduction in orexin in MS patients (either primary or secondary to the CNS demyelinating lesions) could contribute to both the development of neuroinflammation in the disease, and to the presence of sleeping disorders in these patients. Further studies will be necessary to demonstrate a role for endogenously produced orexin in MS.

Orexins have raised as novel therapeutic candidates for sleeping disorders. In order to obtain optimal effects, the development of stable, specific, and potent orexin receptor analogs is critical. A great effort has been put in the discovery of orexin antagonists for the treatment of insomnia [42]. On the contrary, a deficiency of orexin due to degeneration of hypothalamic orexin-producing neurons is associated with narcolepsy, suggesting that orexin A agonists, which are unavailable to date, may potentially exert beneficial actions in this pathology [43]. In addition to narcolepsy, our study suggests that the development of orexin receptor agonists is of great interest for the treatment of inflammatory autoimmune diseases like MS.

## Conclusions

Overall, we have demonstrated that systemic administration of orexin A to mice with established EAE led to a reduction of the clinical symptoms and histopathological features of the disease, globally reducing the inflammatory response in the CNS, without altering T cell encephalitogenic peripheral responses. In addition, orexin A treatment was able to reduce demyelination and glial activation in EAE mice. Given our data, we provided the proof-of-concept that peripheral administration with orexin A may be beneficial in MS.

## Additional files

Additional file 1: **Table S1.** List of primer sets used for PCR and real time RT-PCR. (PDF 64 kb)

Additional file 2: **Figure S1.** Effect of orexin A treatment on myeloid profiles in lymph nodes during the course of EAE. On day 21 post-EAE induction, draining lymph node cells from naive, PBS and OxA groups were analyzed by flow cytometry. Panel A: cells were stained with anti-CD3, anti-CD11b and anti-CD11c antibodies. Representative FACS plots show the percentage of CD3^−^CD11b^+^, CD3^−^CD11c^+^ and CD3^−^CD11b^+^CD11c^+^ subsets. Panel B: cells were stained with anti-CD11b, anti-Ly6C and anti-Ly6G antibodies. Representative FACS plots show the percentage of CD11b^+^Ly6G^+^Ly6C^low^ and CD11b^+^Ly6G^−^Ly6C^high^ subsets. Panel C: histograms show the overlap of CD86, MHCII and PD-L1 between CD11b^+^Ly6G^+^Ly6C^low^ (filled area, top panels) or CD11b^+^Ly6G^−^Ly6C^high^ (filled area, below panels) and CD11b^+^Ly6G^−^Ly6C^−^ (line) subsets. Graphs show the mean percentage of each subpopulation from naïve, PBS and OxA mice (*n* = 7/group). *P < 0.05 (*Mann-Whitney* test, compared to PBS group). (PDF 127 kb)

Additional file 3: **Figure S2.** Orexin A treatment does not impact T cell homeostasis in lymph nodes during EAE. On day 21 post-EAE induction, draining lymph node cells from naive, PBS and OxA groups were analyzed by flow cytometry. Panel A: cells were stained with anti-CD4 and anti-CD8 antibodies. Representative FACS plots show the percentage of CD4^+^ and CD8^+^ subsets. Panel B: cells were stained with anti-CD4, anti-CD8, and anti-Ki67 (proliferative marker) antibodies. Representative histograms of Ki67 for CD4^+^ and CD8^+^ subsets are shown. Panel C: Treg assessment was performed by flow cytometry using a mouse regulatory T cell staining kit. Cells were then stained with anti-CD4, anti-CD25, anti-FoxP3, and anti-Ki67 antibodies. Tregs were defined as CD4^+^CD25^+^FoxP3^+^ cells and proliferative Tregs as CD4^+^CD25^+^FoxP3^+^Ki67^+^ cells. Representative FACS plots show the percentage of Tregs and proliferative Tregs. Panel D: shows Treg profile in draining lymph nodes on day 15 post-immunization. Graphs show the mean percentage of each subpopulation from naïve, PBS and OxA mice (*n* = 7/group). *P < 0.05 (*Mann-Whitney* test, compared to PBS group). (PDF 129 kb)

Additional file 4: **Figure S3.** Orexin A treatment does not modulate the naïve and memory T cell profiles in the draining lymph nodes during EAE. On day 21 post-EAE induction, draining lymph nodes were harvested from naïve, PBS and OxA groups. Cells were analyzed by flow cytometry. In Panel A and Panel B, cells were stained with anti-CD4, anti-CD8 and anti-CD69 antibodies. Representative histograms of CD69 for CD4^+^ and CD8^+^ cells are shown. In Panel C and Panel D, cells were stained with anti-CD4, anti-CD8, anti-CD44, anti-CD62L antibodies. Representative FACS plots of the percentage of CD44^+^CD62L^−^ (effector memory T, Tem), CD44^+^CD62L^+^ (central memory T, Tcm) and CD44^−^CD62L^+^ (naïve T, Tn) subsets are shown. Graphs show the percentage of each subpopulation from naïve, PBS and OxA mice (*n* = 7/group). (PDF 87 kb)

Additional file 5: **Figure S4.** Orexin A does not alter cytokine and chemokine receptor expression in the draining lymph nodes during EAE. The level of cytokine (i.e. IFNγ (Th1), IL-17 (Th17) and IL-4 (Th2)) and chemokine receptor (CXCR3 and CCR6) mRNA expressions were determined by real time RT-PCR in naïve and EAE mice (i.e. 15 and 21 days after EAE induction in PBS- and OxA-treated mice; *n* = 6/group). (PDF 27 kb)

## Abbreviations

Arg-1: Arginase 1
BBB: Blood–brain barrier
BOxA: Before onset orexin A
CFA: Complete Freund’s adjuvant
DLN: Draining lymph node
EAE: Experimental autoimmune encephalomyelitis
GFAP: Glial fibrillary acidic protein
Iba1: Ionized calcium-binding adapter molecule 1
ICV: Intracerebroventricular
IP: Intraperitoneal
IP-10: IFN-γ-inducible protein 10
MBP: Myelin basic protein
MCP-1: Monocyte chemoattractant protein 1
MOG: Myelin oligodendrocyte glycoprotein
MS: Multiple sclerosis
Ova: Ovalbumin
OxA: Orexin A
RO: Retro-orbital
Th: T helper
TLR: Toll-like receptor
Tregs: Regulatory T cells
VIP: Vasoactive intestinal peptide
