# Supplementary material for: Systemic administration of orexin A ameliorates established experimental autoimmune encephalomyelitis by diminishing neuroinflammation
Source: J Neuroinflammation. 2019 Mar 20;16:64. doi: 10.1186/s12974-019-1447-y (PMC6425555; doi:10.1186/s12974-019-1447-y)
Supplement: Supplementary file 1 — Table S1. List of primer sets used for PCR and real time RT-PCR. (PDF 64 kb) [file 12974_2019_1447_MOESM1_ESM.pdf]

# Supplementary Table 1

| Gene | GenBank Accession Number | Primer sequence | |
|---|---|---|---|
| | | Sense | Antisense |
| **Target Gene** | | | |
| **Foxp3** | NM_054039 | 5’-GTATTGAGGGTGGGTGTCAGG-3’ | 5’-GTCAGAGGCAGGCTGGATAAC-3’ |
| **Hcrtr1** | NM_198959 | 5’-GAGCACTGGGCAGATGAACTCT-3’ | 5’-TGCGGAAGATCTGGAAATAGGC-3’ |
| **Hcrtr2** | NM_198962 | 5’-GGTTCATCATCGCCAAGGAGAC-3’ | 5’-TGAGTCGGGTATCCTCATCATAG-3’ |
| **Ifng** | NM_008337 | 5’-GCAACAGCAAGGCGAAAAAG-3’ | 5’-ATCTCTTCCCCACCCCGAAT-3’ |
| **Il4** | NM_021283 | 5’-CGAGGTCACAGGAGAAGGGA-3’ | 5’-AAGCCCTACAGACGAGCTCACT-3’ |
| **Il10** | NM_010548 | 5’-GGTTGCCAAGCCTTATCGGA-3’ | 5’-ACCTGCTCCACTGCCTTGCT-3’ |
| **Il17a** | NM_010552 | 5’-GGAGAGCTTCATCTGTGTCTCTG-3’ | 5’-TTGGCCTCAGTGTTTGGACA-3’ |
| **Ip-10** | NM_021274 | 5’-TGCTGGGTCTGAGTGGGACT-3’ | 5’-CCCTATGGCCCTCATTCTCAC-3’ |
| **Mcp-1** | NM_011333 | 5’-AAAAACCTGGATCGGAACCAA-3’ | 5’-CGGGTCAACTTCACATTCAAAG-3’ |
| **Tgfb1** | NM_011577 | 5’-TGCGCTTGCAGAGATTAAAA-3’ | 5’-AGCCCTGTATTCCGTCTCCT-3’ |
| **Tnfa** | NM_013693 | 5’-GGTGCCTATGTCTCAGCCTCTT-3’ | 5’-CGATCACCCCGAAGTTCAGTA-3’ |
| **Housekeeping Gene** | | | |
| **Hprt** | NM_013556 | 5’-TGGTGAAAAGGACCTCTCGAA-3’ | 5’-TCAAGGGCATATCCAACAACA-3’ |
