# Supplementary figures and images for: Systemic administration of orexin A ameliorates established experimental autoimmune encephalomyelitis by diminishing neuroinflammation
Source: J Neuroinflammation. 2019 Mar 20;16:64. doi: 10.1186/s12974-019-1447-y (PMC6425555; doi:10.1186/s12974-019-1447-y)

# Supplementary Figure 1

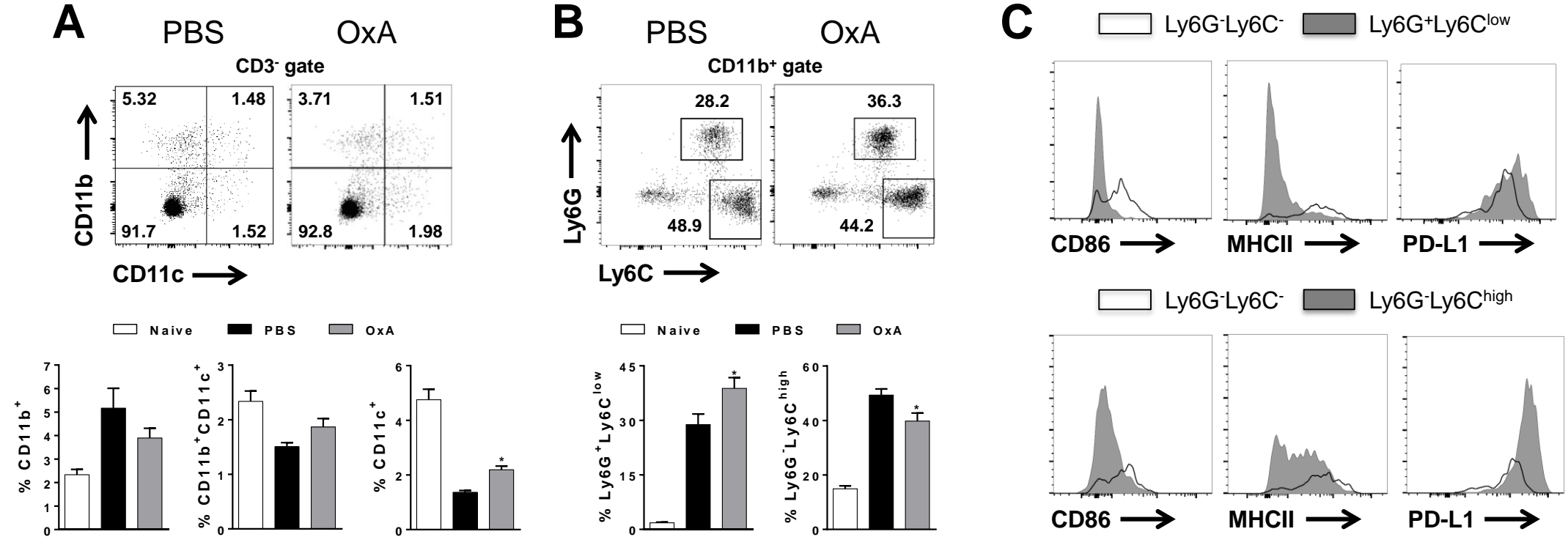

Supplement: Supplementary file 2 — Figure S1. Effect of orexin A treatment on myeloid profiles in lymph nodes during the course of EAE. On day 21 post-EAE induction, draining lymph node cells from naive, PBS and OxA groups were analyzed by flow cytometry. Panel A: cells were stained with anti-CD3, anti-CD11b and anti-CD11c antibodies. Representative FACS plots show the percentage of CD3−CD11b+, CD3−CD11c+ and CD3−CD11b+CD11c+ subsets. Panel B: cells were stained with anti-CD11b, anti-Ly6C and anti-Ly6G antibodies. Representative FACS plots show the percentage of CD11b+Ly6G+Ly6Clow and CD11b+Ly6G−Ly6Chigh subsets. Panel C: histograms show the overlap of CD86, MHCII and PD-L1 between CD11b+Ly6G+Ly6Clow (filled area, top panels) or CD11b+Ly6G−Ly6Chigh (filled area, below panels) and CD11b+Ly6G−Ly6C− (line) subsets. Graphs show the mean percentage of each subpopulation from naïve, PBS and OxA mice (n = 7/group). *P < 0.05 (Mann-Whitney test, compared to PBS group). (PDF 127 kb) [file 12974_2019_1447_MOESM2_ESM.pdf]

# Supplementary Figure 2

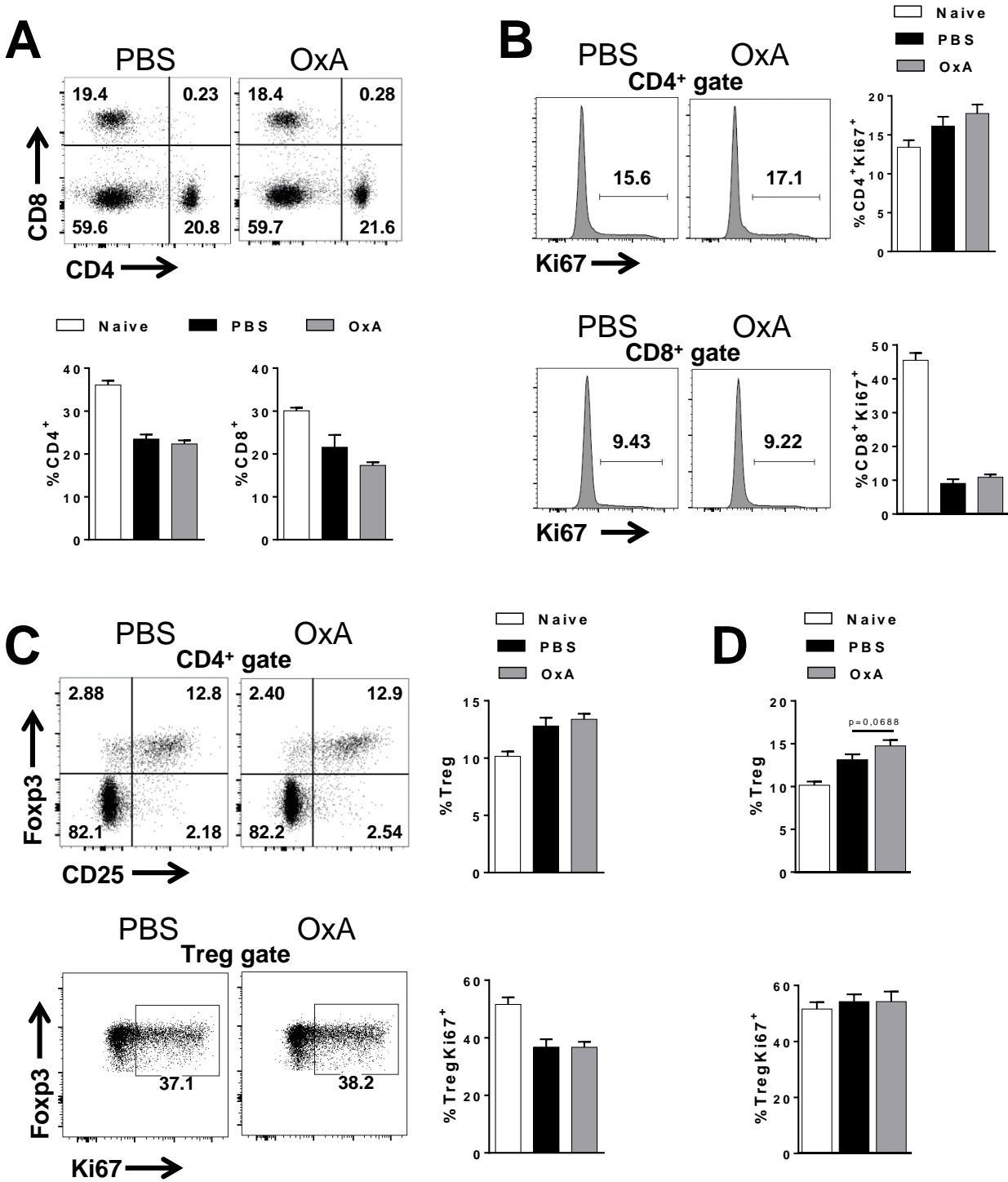

Supplement: Supplementary file 3 — Figure S2. Orexin A treatment does not impact T cell homeostasis in lymph nodes during EAE. On day 21 post-EAE induction, draining lymph node cells from naive, PBS and OxA groups were analyzed by flow cytometry. Panel A: cells were stained with anti-CD4 and anti-CD8 antibodies. Representative FACS plots show the percentage of CD4+ and CD8+ subsets. Panel B: cells were stained with anti-CD4, anti-CD8, and anti-Ki67 (proliferative marker) antibodies. Representative histograms of Ki67 for CD4+ and CD8+ subsets are shown. Panel C: Treg assessment was performed by flow cytometry using a mouse regulatory T cell staining kit. Cells were then stained with anti-CD4, anti-CD25, anti-FoxP3, and anti-Ki67 antibodies. Tregs were defined as CD4+CD25+FoxP3+ cells and proliferative Tregs as CD4+CD25+FoxP3+Ki67+ cells. Representative FACS plots show the percentage of Tregs and proliferative Tregs. Panel D: shows Treg profile in draining lymph nodes on day 15 post-immunization. Graphs show the mean percentage of each subpopulation from naïve, PBS and OxA mice (n = 7/group). *P < 0.05 (Mann-Whitney test, compared to PBS group). (PDF 129 kb) [file 12974_2019_1447_MOESM3_ESM.pdf]

Supplementary Figure 3

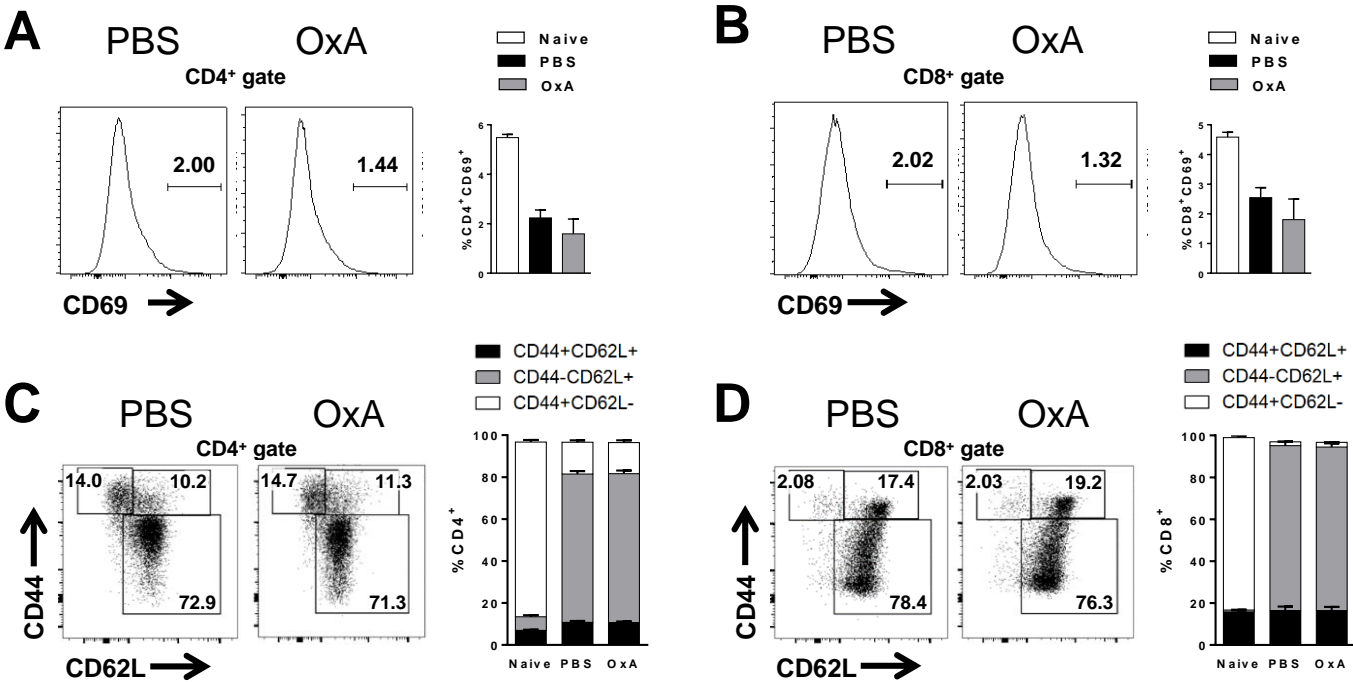

Supplement: Supplementary file 4 — Figure S3. Orexin A treatment does not modulate the naïve and memory T cell profiles in the draining lymph nodes during EAE. On day 21 post-EAE induction, draining lymph nodes were harvested from naïve, PBS and OxA groups. Cells were analyzed by flow cytometry. In Panel A and Panel B, cells were stained with anti-CD4, anti-CD8 and anti-CD69 antibodies. Representative histograms of CD69 for CD4+ and CD8+ cells are shown. In Panel C and Panel D, cells were stained with anti-CD4, anti-CD8, anti-CD44, anti-CD62L antibodies. Representative FACS plots of the percentage of CD44+CD62L− (effector memory T, Tem), CD44+CD62L+ (central memory T, Tcm) and CD44−CD62L+ (naïve T, Tn) subsets are shown. Graphs show the percentage of each subpopulation from naïve, PBS and OxA mice (n = 7/group). (PDF 87 kb) [file 12974_2019_1447_MOESM4_ESM.pdf]

# Supplementary Figure 4

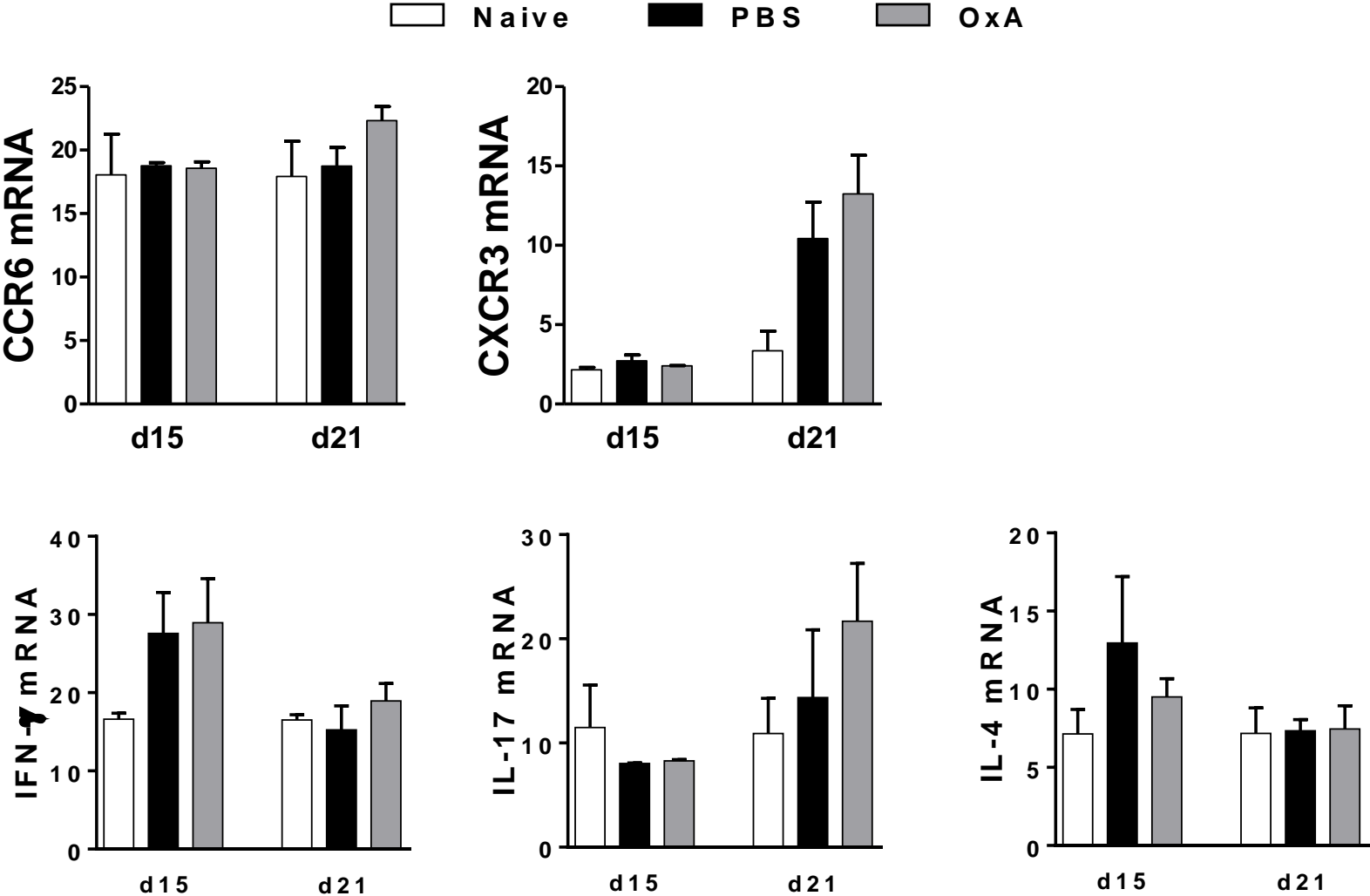

Supplement: Supplementary file 5 — Figure S4. Orexin A does not alter cytokine and chemokine receptor expression in the draining lymph nodes during EAE. The level of cytokine (i.e. IFNγ (Th1), IL-17 (Th17) and IL-4 (Th2)) and chemokine receptor (CXCR3 and CCR6) mRNA expressions were determined by real time RT-PCR in naïve and EAE mice (i.e. 15 and 21 days after EAE induction in PBS- and OxA-treated mice; n = 6/group). (PDF 27 kb) [file 12974_2019_1447_MOESM5_ESM.pdf]
